# Assessment of *Gnaphalium viscosum* (Kunth) Valorization Prospects: Sustainable Recovery of Antioxidants by Different Techniques

**DOI:** 10.3390/antiox11122495

**Published:** 2022-12-19

**Authors:** Stanislava Boyadzhieva, Jose A. P. Coelho, Massimiliano Errico, H. Elizabeth Reynel-Avilla, Dragomir S. Yankov, Adrian Bonilla-Petriciolet, Roumiana P. Stateva

**Affiliations:** 1 Institute of Chemical Engineering, Bulgarian Academy of Sciences, 1113 Sofia, Bulgaria; 2 Instituto Superior de Engenharia de Lisboa, Instituto Politécnico de Lisboa, Rua Conselheiro Emídio Navarro 1, 1959-007 Lisboa, Portugal; 3 Centro de Química Estrutural, Institute of Molecular Sciences, Instituto Superior Técnico, Universidade de Lisboa, Av. Rovisco Pais, 1049-001 Lisboa, Portugal; 4 Department of Green Technology, Faculty of Engineering, University of Southern Denmark, Campusvej 55, 5230 Odense, Denmark; 5 Instituto Tecnológico de Aguascalientes, Aguascalientes 20256, Mexico; 6 CONACYT, Ciudad de México 03940, Mexico

**Keywords:** Mexican Gordolobo, supercritical CO_2_ extraction, phytochemicals, antioxidants, fatty acids, biomass valorization

## Abstract

This work investigates the prospects for exploitation of *Gnaphalium viscosum* (Kunth) abundant but with limited applications till present biomass. The feasibility of traditional techniques (two-phase solvent, and the benchmark Soxhlet extraction) and supercritical extraction without/with a cosolvent at *T* = 40–60 °C and *p* = 30–50 MPa was examined to explore the possibility of recovering phytochemicals from *G. viscosum* leaves, flowers and stems. The efficiency of the techniques was assessed and compared based on yield, influence of solvents used, total phenolic content and antioxidant activity of the extracts. Phenolics of different complexities were identified and quantified by applying LC (LC–MS/MS, and LC–HRAM), while the fatty acid profile was determined by GC–FID. The results of this extensive study demonstrated the huge valorization potential and prospects of *G. viscosum*, since highly potent antioxidants such as kaempferol, kaempferol-3-O-β-d-glucoside (astragalin), and chlorogenic acid were ascertained in considerable amounts. Furthermore, for the first time, the presence of leontopodic acid, a greatly substituted derivative of glucaric acid, was detected in the species.

## 1. Introduction

*Gnaphalium* L. is a genus of flowering plants, commonly called cudweeds, which includes approximately 200 species of the Compositae (Asteraceae) family. It is widespread in temperate and subtropical regions of the world [1,2]. Of the *Gnaphalium* genus, at least 26 species are referred to as “Gordolobo”, also known in English as Mexican Mullein. However, the latter should not be confused with the plant Great Mullein (species *Verbascum Thapsus* L. *Scrophulariaceae* family), which is native to Europe, Africa, and Asia, and is not commonly found in Mexico.

A detailed review, spanning over recent decades, of the *Gnaphalium* genus phytochemical and biological characteristics was published by Zheng et al. [1]. The authors reported that approximately 125 metabolites were identified in the genus comprising, among others, flavonoids, sesquiterpenes, diterpenes, triterpenoids, phytosterols, anthraquinones, acetylenic compounds, carotenoids and some long-chain unsaturated fatty acids. Furthermore, it was shown that extracts of the flowers and leaves of *Gnaphalium* species possess antioxidant, antibacterial, antifungal, anticomplement, antitussive, expectorant, insect antifeedant, cytotoxic, anti-inflammatory, antidiabetic, and antihypouricemic activity.

In another recent review on the application of Mexican plants, Quinones-Bastidas and Navarrete [2] outlined the applications of tea infusion of the inflorescences of the *Gnaphalium* genus in the treatment of asthma, flu, cough, fever and bronchial infections. Attention was also drawn to the fact that at least 10 species of Mexican Gordolobo (MG) have been used for centuries in folk medicine in Mexico and other Latin American counties to treat respiratory ailments and digestive disorders. Mata et al. [3] corroborated and commented that, obviously, the ancient Aztecs were well aware of the medicinal uses of some *Gnaphalium* species.

Some preliminary results about the antimycobacterial activity of MG species on Mycobacterium tuberculosis were reported by Hernández [4]. Another study investigated the antioxidant activity and cytotoxic effect of the flowers and leaf extracts of a particular *Gnaphalium* species (*G. viscosum)* recovered by methanol, *n*-hexane and ethyl acetate, on malignant human cell lines of the cervix and breast [5]. It was speculated that the effect could be a result of the presence of the flavonoid 5-hydroxy-3,7dimetoxi flavone, a compound that helps in the prevention and treatment of various diseases, including cancer.

From the analyses of results published till present, it is evident that extracts of the areal parts of *Gnaphalium* species were mainly obtained by organic solvents (e.g., *n*-hexane, methanol, ethanol, and ethyl acetate) and water. The extract composition was identified by applying different analytical methods. For example, Villagomez-Ibarra et al. [6] examined successive hexane, ethyl acetate and methanol extracts obtained by maceration of air-dried and powdered plant material (flowers, leaves and stems) of three Mexican *Gnaphalium* species. They confirmed the presence of previously isolated constituents such as diterpenoids, flavonoids, and acetylenic compounds, as well as some that are new, and have not been detected before, such as carotenoids, ent-Kaur-16-en-19-oic acid (kaurenoic acid), 13-epi-sclareol, beta-sitosterol, and stigmasterol.

Ontiveros-Rodríguez et al. [7] argued that specialized metabolites of medicinal plants can be considered as their chemical fingerprinting, which is important in order to introduce quality control on medicinal plant species and guarantee the safety of consumption. In view of this, they advocated an NMR-based protocol that can be applied to determine the chemical profiling of commercial samples of MG acquired from different vendors in Mexico City. In order to establish the compositional differences between these samples, special emphasis was placed on the flavones that characterize MG. Extracts from 17 retail samples of MG flowers recovered with a gradient of water:chloroform (1:4, 1:2 and 0:1) in an ultrasonic bath were prepared. The organic phase of the three extracts was analyzed by ^1^H NMR. Formic, malic, gallic, fumaric and malonic acids, the amino acids alanine, asparagine and valine, as well as a complex mixture of sugars were reported. Flavones (e.g., gnaphaliin A, gnaphaliin B, and araneol) were identified by their corresponding ^1^D ^1^H NMR spectra.

From the retrospective analysis of the literature, it can be concluded that plants of the *Gnaphalium* genus, and in particular MG, can be classified as a sustainable resource of vital specialized secondary metabolites with biological activities and prospects for potential applications in modern food, pharmaceutical and other industries, targeting human health and well-being.

As known, the implementation and use of feasible methods and/or intensified technologies towards valorization of renewable biomass is a key factor in the development of sustainable processes. There is a considerable number of conventional and non-conventional methods to isolate secondary metabolites from plants. Still, to the best of our knowledge, till present, as discussed briefly above, the bioactive constituents of different *Gnaphalium*, and MG in particular, species were usually recovered by traditional techniques applying organic solvents. Furthermore, a systematic and in-depth comparison of the influence of the techniques operating parameters on the secondary metabolites content in the extracts obtained is rare. 

Hence, there is a niche, unexplored till present, regarding acquiring insight into the viability, potential, and possible limitations of different methods/techniques—from traditional to sustainable, with low environmental impact, applying green solvents—for extraction of high-quality bioactive compounds from MG species.

Of the different MG species, *Gnaphalium viscosum* (Kunth), an annual or biannual herb, 30–100 cm high, with hairy or downy leaves and inconspicuous flowers, was selected as an object of investigation in the present study. It was not a random choice but one motivated by several important facts—*G. Viscosum* is abundantly present in 27 of Mexico 32 states [7], and can also be found from Canada to Honduras, as well as in Northern South America; is among the species mainly considered as MG by the Mexican Herbal Pharmacopoeia [8]; has demonstrated great potential as an antibacterial agent [2,6]; its cultivation is not limited by any specific requirements or inclusions in protected species lists, and obtaining its biomass would require minimum investment. In view of the above, the main aim of our research was to explore the potential of three techniques to recover secondary metabolites—antioxidants and fatty acids—from the leaves, flowers and stems of *G. viscosum.* To achieve that goal, the efficiency and sustainability of the techniques were compared on the basis of extraction yield, composition and quality of extracts recuperated.

The first two methods are conventional extractions applying organic solvents. The third one is extraction with supercritical CO_2_ (scCO_2_), either neat or with a co-solvent. Supercritical extraction (SCE) is considered to be among the most sustainable green alternatives to the conventional ones. Its particular benefits include no waste production, shorter extraction time, automation, lower solvent consumption, and no presence of organic solvents in the extracts. Of particular importance for the recovery of heat-sensitive compounds are the low-temperature operative conditions of SCE, as prolonged heating during the removal of solvent could lead to degradation of secondary metabolites [9].

To the best of our knowledge, the application of neat scCO_2_ and scCO_2_ with a co-solvent to the recovery of bioactives from *G. viscosum* is unique. Moreover, in the open literature, we only found one article devoted to the SCE of Mullein species, namely extraction of dried areal parts of Common mullein (*Verbascum thapsus* L.) by scCO_2_ at 30 MPa and 313.2 K with the view to determine the antibacterial activity of the extracts [10].

The composition of phenolics in representative extracts was identified and quantified by LC (liquid chromatography high-resolution accurate mass—LC–HRAM, and liquid chromatography with tandem mass spectrometry—LC–MS/MS) analyses, while the fatty acid profile was determined by GC–FID. Furthermore, total phenolic content was measured, and the antioxidant activity of the extracts was determined by ABTS and DPPH.

On the basis of the results obtained, it was possible to critically analyze the influence of the techniques’ specifics (operational parameters and application of given solvents/co-solvents) on the quality of the extracts recovered. Subsequently, that info can be used to reveal *G. viscosum* valorization prospects and potential for its efficient utilization in pharmaceutical, food, cosmetic, and other industries.

As far as we are aware, the present research, which combined the concerted efforts of scientists from four countries, is the first to report the application of conventional methods and a supercritical fluid extraction technique to the recovery and analyses of secondary metabolites derived from the MG species *G. viscosum* (Kunth).

## 2. Materials and Methods

### 2.1. Plant Material

The species *Gnaphalium Viscosum* (Kunth) biomass was purchased from a local herb pharmacy in Aguascalientes, Mexico. Gordolobo material was separated by hand into leaves, flowers, and stems. Each fraction was grinded in a household blender (Heinner, Bucharest, Romania). The average particle diameter of the material, subjected to further extractions, was determined to be less than 1 mm.

### 2.2. Chemicals and Reagents

The main standards used in the analyses of phenolic compounds by LC–MS/MS, and all other relevant data were presented in detail in a previous work [9]. The additional standards were leontopodic acid A, cat. N 6026S, and leontopodic acid B, cat. N 6032S. 

Chemicals applied for TPC, antioxidant activity assays (ATBS and DPPH): ethanol HPLC grade (Panreac, Barcelona, Spain), gallic acid, Folin–Ciocalteu reagent 2 N, Trolox (6-hydroxy-2,5,7,8-tetramethylchroman-2-carboxylic acid), DPPH• (2,2-diphenyl-1-picrylhydrazyl) (Sigma-Aldrich, St. Louis, MO, USA), sodium carbonate (Merck, Darmstadt, Germany), potassium persulfate and absolute ethanol (Neon, Suzano, SP, Brazil), and quercetin dihydrate (Sigma-Aldrich Chemie GmbH., Steinheim, Germany).

Chemicals used for the GC–FID analyses: Supelco 37 Component FAME Mix (CRM47885), toluene (pure, VWR International, France), sulfuric acid (98%, MerckKGaA, Germany), sodium chloride (pure, MerkKGaA, Darmstadt, Germany), potassium bicarbonate (pure, VWR International, Paris, France), chloroform (99.8% VWR International, Paris, France), sodium sulfate (pure, Sigma-Aldrich Chemie GmbH, Taufkirchen, Germany), and helium (99.9999%, Air Liquide A/S).

The rest of the reagents applied were of the highest purity: methanol ≥ 99.9%, ethanol ≥ 99.8% and *n*-hexane ≥ 99% were purchased from Honeywell Riedel-de-Haen (Seelze, Germany), ethyl acetate ≥ 99.5% from JLS-Chemie Handel GmbH (Hannover, Germany), methyl tert-butyl ether ≥ 99.8%, and acetonitrile ≥ 99.9% from Sigma-Aldrich (Darmstadt, Germany) and bone dry grade CO_2_ (99.99% pure; No water, Messer, Sofia, Bulgaria).

### 2.3. Two-Phase Solvent Extraction

From each of the three samples, 1.00 g of material was weighed by an analytical balance, and to each of them 25 mL of solution A (water/methanol (3/1, (*v/v*)) and 15 mL of solution B (methyl tert-butyl ether/methanol (3/1, vol.)) were added. Each mixture was stirred in a mechanical homogenizer for 1 min at 36000 rpm and at room temperature. Consequently, the mixture was stirred in a rotator for 24 h, after which the samples were centrifuged for 20 min at 6500 rpm and at 4 °C.

The upper (top) layer containing non-polar substances and chlorophyll was removed and subsequently evaporated to dryness in a rotary vacuum evaporator at a temperature below 35 °C. All samples produced an oil-resembling product.

The bottom water–alcohol layer was filtered on a paper membrane; the solid mass was washed three times with 10 mL of solution A. Then, the combined filtrate was evaporated to dryness on a rotary evaporator at a temperature below 40 °C. The resulting solid residue was dissolved in 10% acetonitrile, and lyophilized. 

The extracts were subsequently analyzed by LC–HRAM and LC–MS/MS.

### 2.4. Soxhlet Extraction

All experiments were performed by means of the Soxhlet apparatus ISOLAB NS29/32+34/35 (Merck KGaA, Darmstadt, Germany). Three solvents with different polarities (*n*-hexane, ethyl acetate and ethanol) were used (Table 1). The ratio between the liquid: solid phases was 30:1.

In all experiments, the extraction cartridge was filled with 7.0 ± 0.1 g dry material (*G. viscosum* leaves, flowers or stems). The extraction time was different and depended on discoloration of the solvent. After each extraction, the solvent from the liquid extract was evaporated under vacuum using a Hei-VAP Rotary Evaporator (Heidolph Instruments GmbH&Co. KG, Schwabach, Germany).

The resulting dry extract was further dried to a constant weight in an air circulation oven, at 333.15 ± 2.0 K, and the yield was evaluated according to Equation (1):

(1) $$\mathrm{Yield}\left(\%\right)=\frac{\mathrm{massofextract}\;\left(g\right)}{\mathrm{massofsample}\;\left(g\right)}*100$$

The extracts obtained were placed in glass vials and kept at 4 °C until analysis by LC–MS/MS and GC–FID. Experiments were performed in triplicates and total extraction yield was expressed as the mean ± standard deviation.

### 2.5. Supercritical Fluid Extraction (SFE)

In our study, the SFE experiments were performed in a flow apparatus (SFT-110-XW, Supercritical Fluid Technologies Inc., Newark, DE, USA), equipped with two parallel 50 cm^3^ internal volume extractors made from stainless steel tubing (7 cm long, internal diameter 3.02 cm) and temperature controllers for extraction vessels and restrictor valves, which can be adjusted up to 393.2 K.

The required pressure of the CO_2_ from the tank (room temperature) is ensured by a SFT Nex10 SCF pump actuated from a compressor model HYAC50-25, Hyundai, Seoul, Republic of Korea. The maximum pressure is 60 MPa.

The CO_2_ flow rate at the outlet of the extraction cell is measured by a flow meter and a totalizer from Alicat Scientific (Tucson, AZ, USA), model M-5SLPM-D/5M. In experiments with a co-solvent, an additional pump (LL-Class, State College, PA, USA) is used.

The extraction with neat scCO_2_ of *G. viscosum* biomass was performed at *T* = (313.2, 323.2 and 333.2) K and *p* = (30, 40 and 50) MPa. The scCO_2_ flow rate was 1.9 × 10^−3^ kg⋅min^−1^. For the extractions carried out using CO_2_ with a co-solvent, ethanol, the two pumps (for CO_2_ and the co-solvent) were adjusted so that the final value of the CO_2_ flow rate was 1.9 × 10^−3^ kg⋅min^−1^. The values in percentage of the co-solvent were (5% and 10%) mol fractions, accordingly.

In all experiments, approximately 5 g dry sample of the respective matrix section of the *G. viscosum* plant biomass was placed in the processing vessel. The bottom and top of the extractor contain two metal frits (2 µm), and its lower part was filled with propylene wool, which was also placed at the top to avoid the entrainment of any material. The uncertainties of the temperature and pressure measurements were 1 °C and 0.1 MPa, respectively. The gravimetric measurements were performed on an analytical balance with an uncertainty of 0.1 mg and with a coverage factor of 2.

Once the system has equilibrated at the selected pressure and temperature, the static/dynamic valve on the oven is opened following the restrictor valve opening to achieve the required flow rate of liquid carbon dioxide through the system and the dynamic extraction takes place.

The extract fractions, without replicates, were collected at ambient pressure into glass vials, placed in an ice bath. The vials were changed every two minutes until no extract was collected in two consecutive vials. For the cases of SFE with a co-solvent, the solvent was evaporated in an air circulation oven at 338.2 K until constant weight.

The samples were kept at 277.2 K in the dark until analysis with GC–FID (neat scCO_2_) and LC–MS/MS (with ethanol as a co-solvent).

### 2.6. Characterization and Quantification of the Extracts

#### 2.6.1. LC–High-Resolution Accurate Mass analysis (LC–HRAM)

LC–HRAM analysis is a powerful tool that allows detection of complex analytes at low concentrations and accurate identification of components. In our case, the analyses were carried out on a Q Exactive^®^ hybrid quadrupole-Orbitrap^®^ mass spectrometer (Thermo Scientific Co., Waltham, MA, USA) equipped with a HESI^®^ (heated electrospray ionization) module, a TurboFlow^®^ Ultra High-Performance Liquid Chromatography (UHPLC) system (Thermo Scientific Co., Waltham, MA, USA) and a HTC PAL^®^ autosampler (CTC Analytics, Zwingen, Switzerland).

##### Chromatographic Conditions

The chromatographic separations of the analyzed compounds was achieved on a Nucleo shell C18 (100 × 2.1 mm, 2.7 µm) analytical column (Macherey-Nagel, Germany), using gradient elution at a 300 µL/min flow rate. The eluents used were: A—0.1% formic acid in water; B—0.1% formic acid in ACN. The following binary gradient was used: Start at 0% B, hold for 2 min; 0–40% B—26 min, 40–90% B—3 min; 90% B—1 min; 90–0% B for 2 min and 0% B for 3 min.

##### Mass Spectrometry Conditions

Full-scan mass spectra over the m/z range 100–1200 were acquired in the negative ion mode (NIM) at resolution settings of 70,000. Parallel reaction monitoring (PRM) mode at resolution settings of 17,500 and 0.5 amu isolation window of precursor ions was used for quantitative analysis. Qualitative analyses were carried out using AIF (all ion fragmentation), top N (5) and PRM scans of operation of mass analyzer in the negative mode.

The mass spectrometer operating parameters used in the negative ionization mode were as those described in detail in a previous work [9], with the following changes: capillary temperature—320 °C; probe heater temperature—300 °C; auxiliary gas flow 12 units; sweep gas 2 units (units refer to arbitrary values set by the Q Exactive Tune software) and S-Lens RF level of 50.00. All derivatives were quantified using 5 ppm mass tolerance filters to their theoretical calculated m/z values. Data acquisition and processing were carried out applying the software package (Thermo Scientific Co., Waltham, MA, USA), as reported in [9].

##### Quantitative Analysis

Standards available at the lab were used. The results obtained are based on external calibration and use of PRM in the negative mode of operation of the mass analyzer, and represent the mean values based on 3 replicates (independently processed samples).

#### 2.6.2. Liquid Chromatography with Tandem Mass Spectrometry (LC–MS/MS) Analysis

The methodology of quali-quantification of phenolics was explained in detail in an earlier work [9]. Here, just the main steps involved are summarized.

Standard and sample preparations were analogous to those reported in [9].

The LC–MS/MS analyses were carried out on a Q Exactive^®^ hybrid quadrupole-Orbitrap^®^ mass spectrometer (Thermo Scientific Co., Waltham, MA, USA) equipped with a HESI^®^ (heated electrospray ionization) module, a TurboFlow^®^ Ultra High-Performance Liquid Chromatography (UHPLC) system (Thermo Scientific Co., Waltham, MA, USA) and a HTC PAL^®^ autosampler (CTC Analytics, Switzerland).

##### Chromatographic Conditions

The chromatographic separations of the compounds were performed on a Nucleodur C18 Gravity (100 × 2.1 mm, 1.8 µm) analytical column (Macherey-Nagel, Düren, Germany) using gradient elution at a 0.3 mL⋅min^−1^ flow rate, and eluents A—0.1% formic acid in water; B—0.1% formic acid in ACN (please see above).

Mass spectrometry conditions: Full-scan mass spectra over the m/z range 100–1200 were obtained in NIM at resolution settings of 70,000. The PRM mode was analogous to that of LC–HRAM analyses.

The operating parameters of the mass spectrometer were those reported in [9]. The quantification of the compounds identified was performed as also described in [9], and the results represent the mean values based on 3 replicates (independently processed samples).

#### 2.6.3. Gas Chromatography (GC) Analysis

The nature and profile of the fatty acids in certain extracts of *G. viscosum* recovered by Soxhlet *n*-hexane and neat scCO_2_ were determined by Gas Chromatography—Flame Ionization Detection (GC–FID) of methyl esters (FAME). The methodology is described in detail in [11]. In brief:

##### Sample Preparation

In a test tube, approximately 5 mg of the sample was dissolved in 1 mL toluene. A volume of 2 mL of 1% sulfuric acid in methanol was added and left overnight at 50 °C. A volume of 5 mL of sodium chloride (5%) solution was added before the esters were extracted with hexane (2 × 5 mL) and layers separated. The hexane layer was transferred to a clean test tube and washed with 4 mL of potassium bicarbonate (2%) solution. The hexane layer was then dried over anhydrous sodium sulfate. The solution was filtered through a 0.45 μm syringe filter and the solvent removed with a rotary evaporator under vacuum. The dried extract was redissolved in 1 mL hexane and 0.250 mL of chloroform before analysis.

##### Chromatographic Conditions

Samples were run on a GC Agilent 7890B equipped with an FID detector and a Agilent J&W DB-FFAP column (30 mm × 0.032 mm × 0.25 μm), with an injection volume of 1 μL. Split/splitless injection mode, 280 °C, split ratio 50:1, carrier gas—helium, 42 cm/s, constant flow mode. 

The temperature gradient of the oven was set to: 120 °C (2 min), 5 °C/min to 140 °C (3 min); 20 °C/min to 250 °C (10 min). The FID was operating at 280 °C, hydrogen: 40 mL/min; air: 400 mL/min; make-up gas: 25 mL/min 

Identification was performed based on the different retention times of the analytes. Analyses were performed in triplicate and the results are presented as the relative percent of each fatty acid in each of the samples analyzed.

#### 2.6.4. Measurement of Total Phenolic Content

Total phenolic content (TPC) of all analyzed samples was determined using the Folin–Ciocalteu reagent [12]. In brief, a 20 µL aliquot of each sample was mixed with 100 µL Folin–Ciocalteu phenol reagent and 300 µL freshly prepared Na_2_CO_3_ (15% (*w*/*v*)) and was incubated for 2 min at room temperature. The reaction mixtures were completed to a final volume of 2 mL with deionized water, vortexed and incubated further for 2 h at room temperature. A 200 µL aliquot from each sample was transferred to a 96-well plate and the absorbance was measured at 765 nm using Varioskan multiplate reader (Thermo Electron Corporation, Vantaa, Finland). A calibration curve was obtained using the standard quercetin (0–700 mg/L) and the results were expressed as mg quercetin equivalents/L (mg QE/L) of sample. All determinations were carried out in triplicates and the results are expressed as the mean values.

#### 2.6.5. Antioxidant Activity

##### DPPH Assay

The free radical scavenging activity of the samples was determined by the DPPH assay as previously described [13]. The DPPH solution (0.1 mM) was freshly prepared in methanol and 980 µL of the solution were mixed with a 20 µL aliquot from each analyzed sample. Methanol was used as the negative control. The reaction mixture was incubated for 1 h at room temperature in the dark and the absorbance was measured at 518 nm using a Varioskan multiplate reader. A calibration curve was obtained using Trolox (0–1.0 mM) and the results were expressed as mM Trolox equivalents (mM TE). All determinations were carried out in triplicates and the results are expressed as the mean values.

##### ABTS Assay

ABTS radical scavenging activity was determined by direct absorbance measurement of the radical (ABTS+) [14]. The radical was generated by mixing 2.5 mL of ABTS (7 mM solution in water) with 44 µL of 140 mM potassium persulfate. The solution was kept in the dark for 12–16 h till the development of a blue-green color, and diluted with 70% methanol to final absorbance of 0.700 ± 0.020 at 734 nm. The solution was used on the same day by mixing 200 µL of the diluted ABTS+ with 5 µL of fresh standard (0–1.0 mM Trolox) or sample. After 5 min, the absorbance was measured at 734 nm, using methanol as blank. The results were expressed as mM Trolox equivalents (mM TE). Determinations were performed in triplicates and expressed as the mean values.

## 3. Results and Discussion

This section is organized in the following way: firstly, the extraction techniques’ efficiency assessed based on the yields achieved is analyzed and compared. The impact of operating parameters (solvents, temperature and pressure) is also discussed. Then, the chemical composition, total phenolic content and antioxidant activity of chosen flowers, leaves, and stem extracts recovered by the three extraction methods and identified and quantified by the analytical methods employed are presented and compared.

### 3.1. Extraction Yield

For the two-phase solvent extraction, the yields of the lyophilized dry matter were as follows: leaves—134.5 mg, flowers—110. 3 mg, stems—86.7 mg or (13.45, 11.03, 8.67)%, respectively.

Extraction yields obtained by Soxhlet with the three solvents, with polarity relative to water of 0.654, 0.228, 0.09 (ethanol, ethyl acetate and *n*-hexane), respectively [15], are displayed in Table 1.

**Figure 1:**
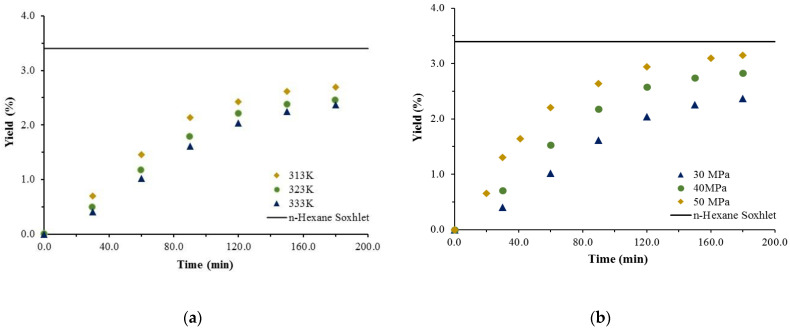
Cumulative experimental kinetic extraction curves plotted vs. the extraction time, at a scCO_2_ flow rate of 1.9 × 10^−3^ kg/min. Influence of temperature at a constant pressure of 30 MPa (**a**) and pressure at a constant temperature of 333 K (**b**) on the yield for *G. viscosum* flowers.

**Figure 2:**
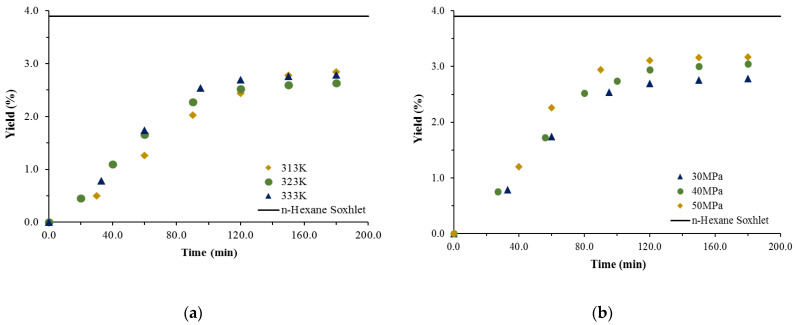
Cumulative experimental kinetic extraction curves plotted vs. the extraction time, at a scCO_2_ flow rate of 1.9 × 10^−3^ kg/min. Influence of temperature at a constant pressure of 30 MPa (**a**) and pressure at a constant temperature of 333 K (**b**) on the yield for *G. viscosum* leaves.

The extractions with neat scCO_2_ were performed on *G. viscosum* flowers and leaves only. The reason behind that was that the yield of the *n*-hexane Soxhlet extraction of the stems fraction was quite low, and typicallyscCO_2_ yield is lower than the former. The SCE temperature was varied in the range 313.2–333.2 K, and pressure in the range 30–50 MPa. Representative cumulative experimental kinetic extraction curves were plotted to assess the effect of operating conditions (at a constant flow rate) on the yield, as well as a comparison with the Soxhlet *n*-hexane extraction yield (represented by the continuous line, parallel to the abscissa), are shown on Figure 1 and Figure 2 for the flowers and leaves, respectively.

The impact of the temperature on the extraction yield (Figure 1a and Figure 2a) is not definitive in the sense that at lower temperatures, despite an increase in the yield, it was quite insignificant. Yield decrease with the rise in temperature was discussed by Coelho et al. [16], who explained that in part with the balance between two opposite effects: increasing the temperature decreases the density of the scCO_2_ and thus its solubility capacity; but at the same time, it increases the vapor pressure of the compounds, consequently enhancing their solubility in the supercritical fluid. The influence of pressure, on the other hand, is in all cases positive, clearly demonstrated and straightforward—at a given temperature increasing the pressure improves the yield (Figure 1b and Figure 2b). Thus, the highest yields for the flowers and leaves—3.1% and 3.17%, respectively—are achieved at the highest pressure applied and, though still lower, they are almost commensurable with that of *n*-hexane Soxhlet (3.48%). Moreover, it should be noted that 80% of the extract in both cases is recovered for a much shorter time than that required by the Soxhlet *n*-hexane.

With regard to the influence of solvents/co-solvents, the highest yields for the Soxhlet extractions were achieved by the solvent with the highest polarity among those examined—ethanol, followed by ethyl acetate, and *n*-hexane. For the SCE, experiments were performed with 10% ethanol on the three areal parts of *G. viscosum*. In addition, scCO_2_ with 5% ethanol was applied to the leaves only, as their yield with Soxhlet ethanol was the highest among all measured.

**Figure 3:**
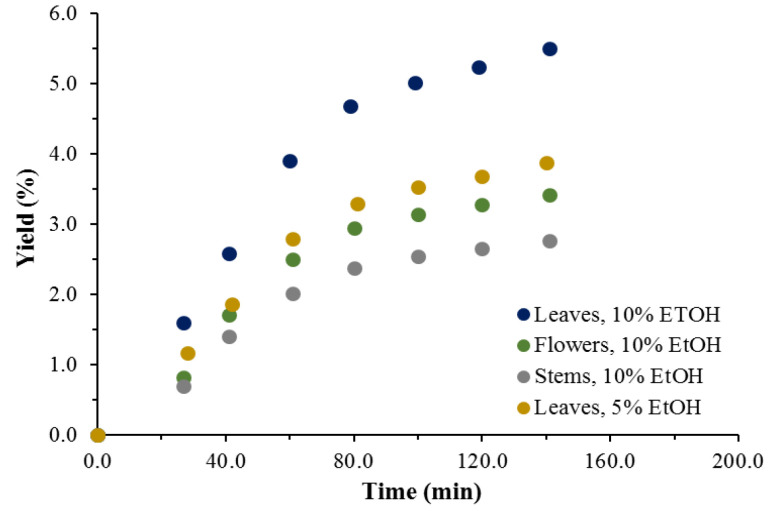
Cumulative experimental kinetic extraction curves plotted vs. the extraction time, at a scCO_2_ flow rate of 1.9 × 10^−3^ kg/min, *T* = 333 K, *p* = 50 MPa. Influence of the co-solvent ethanol on the extraction process.

The cumulative experimental extraction curves plotted to assess the effect of the addition of a co-solvent to scCO_2_ at the previously determined most favorable towards the yields values of the operation parameters temperature and pressure are shown in Figure 3.

The scCO_2_ + ethanol extraction process was realized for approximately 30% less time than that required with neat CO_2_, at the same flowrate. Nevertheless, the yields achieved are still considerably lower than those of Soxhlet ethanol. For example, the highest yield for the leaves is approximately 3-fold lower than that of the latter but is obtained with a much lower amount of ethanol (only 10% in the composition of the solvent), and at a much shorter operation time. The scCO_2_ + ethanol yields are commensurable with Soxhlet ethyl acetate yields, e.g., 2.76 vs. 2.88% for the stems.

The two-phase solvent extraction yields for the leaves and flowers, respectively, are lower than the corresponding ones of Soxhlet ethanol. However, for the stems, this technique renders a slightly higher yield than that of Soxhlet ethanol (8.6 vs. 8.2%), respectively, and for all three *G. viscosum* fractions its yields are higher than those of scCO_2_ +ethanol. Still, the latter surmounts important limitations of conventional extractions as it uses less organic solvent and does not cause any harm to the environment.

### 3.2. Phytochemical Analysis

#### 3.2.1. Analysis and Quantification of Antioxidants

Firstly, extracts of the two-phase solvent technique were analyzed by LC–HRAM. Prompted by a previous work [17], the analyses were concentrated on demonstrating whether presence of derivatives of caffeoylquinic, and in particular caffeoyl-D-glucaric acids would be detected. All samples were analyzed in identical concentrations and at identical analytical conditions.

**Figure 4:**
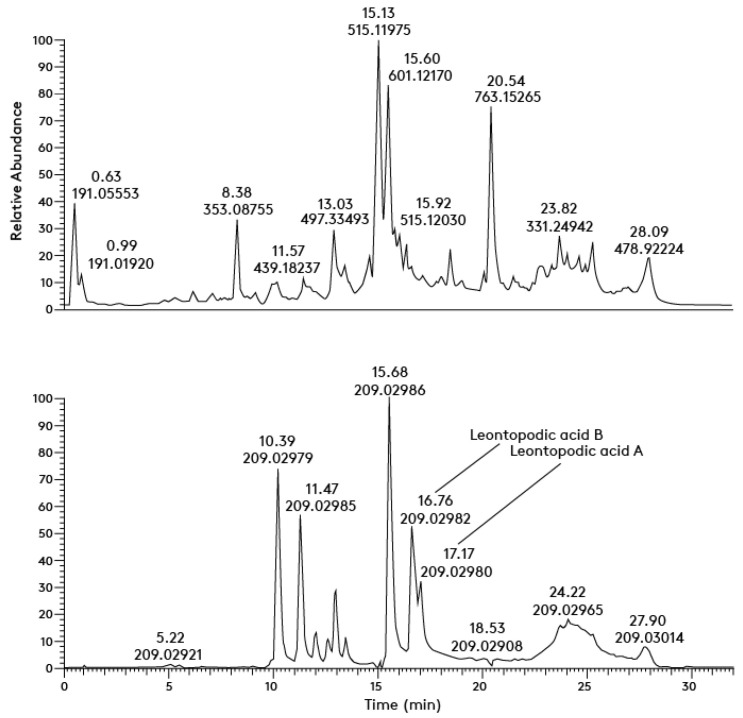
Mass chromatogram of compounds containing MS/MS fragment ion [M − H]− = 209.023 specific to substances comprising caffeoyl-D-glucaric acids.

The results obtained are illustrated by a mass chromatogram for the leaf extracts only. Thus, Figure 4 displays an exemplary mass chromatogram of compounds containing MS/MS fragment ions specific to substances comprising caffeoyl-D-glucaric acids. For the flower and stem extracts, the picture is identical with some variations.

Quantitative analyses were performed for 3-O-caffeoylquinic (chlorogenic) and 5-O-caffeoylquinic (neo-chlorogenic) acids, and for the highly substituted glucaric acid derivatives leontopodic acids A and B, using standards available at the laboratory. The results obtained are based on external calibration and use of PRM in the negative mode of operation of the mass analyzer and are displayed in Table 2.

The highest quantity of chlorogenic acid is registered in the flowers, and the lowest in the stems. The amounts of the neo-chlorogenic acid are much lower and follow the same trend. The quantities of leontopodic acids A and B are the highest in the stems and lowest in the leaves. However, the amounts of leontopodic acid B are much higher, e.g., in the stems, its quantity is over 80-fold higher than that of leontopodic acid A.

Subsequently, in order to obtain a deeper knowledge and improved comprehension about the extract composition, particularly regarding the presence of phenolics of different chemical complexities, certain extracts of *G. viscosum* flowers, leaves and stems recovered by the three techniques applied were analyzed by LC–MS/MS.

The results obtained are displayed in Table 3 and Table 4, respectively.

The quali-quantification of the extracts demonstrate that the three areal parts of *G. viscosum* are rich in important multifunctional ingredients, belonging to the hydroxycinnamic, caffeoylquinic, and hydroxybenzoic acids, respectively, and to several flavonoid subgroups, etc. In the recuperated by the different techniques *G. viscosum* extracts though, the quantities of the bioactives vary sometimes by orders of magnitude, which demonstrates the influence of the recovery methods operating conditions and solvents applied.

Chlorogenic (3-O-caffeoylquinic) acid is by far the most abundant among all acids in the extracts recovered by the two-phase solvent extraction and Soxhlet ethanol. In the flower extracts of the latter, chlorogenic acid quantity is approximately 1.5 higher than that in the corresponding extract of the first technique, while the amounts in the leaves and stems are lower and commensurable for both techniques. A dramatic change in the quantities of chlorogenic acid is observed for the scCO_2_ + ethanol extracts. Though the highest amount is still found in the flowers, it is approximately 90-fold lower than that recovered by Soxhlet ethanol and over 57-fold lower than the one of the two-phase solvent extraction. Obviously, the change in the chlorogenic acid amounts registered in the scCO_2_ + ethanol extracts can be an indication of the fact that the quantity of the co-solvent ethanol applied is far from sufficient to recover chlorogenic acid in full. That is further supported by the results obtained for the leaf extracted with 5% ethanol—the quantity of chlorogenic acid is over 21-fold lower than in the leaves but recuperated by 10% ethanol.

The demonstrated richness in chlorogenic acid of the flower, leaf and stem extracts obtained by the two-phase solvent and Soxhlet ethanol provides valuable information that can lead to new prospects for their use for health benefits, since chlorogenic acid has proven antidiabetic, anticarcinogenic, anti-inflammatory and antiobesity impacts [18].

Another interesting characteristic of the scCO_2_ + ethanol flower, leaf and stem extracts is that caffeic acid is now dominant among all in the hydroxycinnamic and caffeoylquinic acid derivatives group. Moreover, its quantity in the flower extract is the highest among all acids of the two groups—and for comparison, is approximately 334-fold higher than that recovered by Soxhlet ethanol (1999.37 vs. 5.99) ng/mg. Ferulic and *o*-coumaric acids are the second and third most abundant acids in the flowers—a trend similar to that exhibited by the Soxhlet ethanol and the two-phase solvent extracts but their quantities are by an order of magnitude lower, e.g., 206.24 vs. 14.01 ng/mg ferulic acid in the scCO_2_ + ethanol vs. Soxhlet ethanol flower extracts. In the leaves and stems, the quantities of ferulic and *o*-coumaric acids for the three techniques are in the same range of magnitude.

An intriguing and a completely different picture is obtained for the hydroxybenzoic acids recuperated by the three techniques. Though, in all extracts, gentisic acid is in the highest amounts, its quantities in the scCO_2_ + ethanol flower extract are approximately 1.5-fold higher than Soxhlet ethanol and over 3-fold higher than that of two-phase solvent extraction. For the leaves and stems, however, the trend is reversed; the quantities of gentisic acid in the Soxhlet ethanol extracts are higher than those of scCO_2_+ ethanol, the latter being commensurable with those of the two-phase solvent.

scCO_2_ + ethanol extraction demonstrates a steady trend in the recovery of vanillic, *o*-hydroxybenzoic, syringic and 3-OH-4-methoxybenzoic acids. Their quantities in the flower, leaf and stem extracts are much higher than those recuperated by the two-phase solvent and Soxhlet ethanol, respectively. The only exception is the amount of 3-OH-4-methoxybenzoic acid detected in the stems extract of the former. Moreover, if compared to Soxhlet ethanol, it shows higher selectivity regarding the above acids.

The results obtained reveal that for the flowers, scCO_2_ + ethanol, at the operating conditions tested, exhibits much higher selectivity towards certain hydroxybenzoic acids and promotes their recovery in higher amounts, when compared to the two-phase solvent and Soxhlet + ethanol. For the leaves, and stems, however, there is not a steady clear trend demonstrated, gentisic acid being the most prominent example—its quantities in the extracts recovered by Soxhlet ethanol are higher than those registered in scCO_2_ leaf and stem extracts, respectively.

A closer examination supports the assumption that the amounts of the acids obtained depend not only on the matrix (flowers vs. leaves vs. stems), but on the extraction method, and in particular solvents applied.

In addition to the acids groups, several subgroups of the complex secondary metabolites belonging to the Flavonoids family were detected. Among those, the most remarkable by far is the flavonols subgroup.

A notable feature of *G. viscosum* is the presence of kaempherol-3-O-β-d-glucoside, known as astragalin, in very high quantities in all extracts recovered by the two-phase solvent and Soxhlet ethanol. The highest amount was identified in the flowers, followed by the leaves and stems for both techniques. Kaempherol-3-O-β-d-glucoside quantities in the extracts of the two-phase solvent and Soxhlet ethanol are not only the highest in the flavonols subgroup, but the highest among all secondary metabolites identified and quantified in the extracts obtained by the three techniques. Moreover, the amount of 76,129.67 ng/mg detected for kaempherol-3-O-β-d-glucoside in the Soxhlet ethanol flower extract is the absolute maximum among all phenolics identified and quantified by the three techniques.

While the quantities of that compound in the extracts recovered by the two-phase solvent are lower, still they do not differ by orders of magnitude—76,129.67, 7993.67, and 9827.97 ng/mg vs. 45,999.92, 6589.52, and 1207.40 ng/mg—when compared to Soxhlet ethanol, which is in a striking contradiction with the amounts registered in the scCO_2_ + ethanol extracts—2574.92, 221.52, and 45.81 ng/mg. It should also be noted that kaempherol-3-O-β-d-glucoside quantities in the Soxhlet ethanol stem extracts are higher than those in the leaves, a tendency not observed for the extracts of the two-phase solvent and scCO_2_ + ethanol. Moreover, the amount of astragalin in the Soxhlet ethanol stems extract is in the second place among all flavonoids and is over 8-fold higher than that in the extract of the two-phase solvent.

The other bioactives in relatively high amounts in the Soxhlet ethanol extracts are myricitrin, kaempferol, and quercetin (in diminishing amounts in that order). The trend observed for the two-phase solvent extraction is almost analogous but the quantities of the first two compounds in the line are commensurable.

For the extracts of scCO_2_ + ethanol, as mentioned previously, the picture is completely different: now kaempferol, and not kaempherol-3-O-β-d-glucoside whose quantities have diminished to the fourth place following those of kaempferol, naringenin and quercetin, is the most abundant in the flavonols group. In addition, kaempferol amount is by far the highest among all phenolics identified and quantified in the scCO_2_ + ethanol extracts, and is higher than that detected in the two-phase solvent and Soxhlet ethanol extracts (e.g., in the flowers is approximately 10-fold higher than that recovered by Soxhlet ethanol). The quantity of quercetin is the second highest. It is over 7.5-fold higher than the corresponding that in Soxhlet ethanol, which performs better than the two-phase solvent regarding that compound.

Kaempferol and kaempherol-3-O-glucoside are very important antioxidants. Many studies have described the beneficial effects of dietary kaempferol in reducing the risk of chronic diseases, especially cancer, as it inhibits cancer cell growth and angiogenesis and acts as a powerful promoter of apoptosis, while preserving normal cell viability [19]. Furthermore, kaempferol has a role as an antibacterial agent, a human xenobiotic, and blood serum metabolite, a human urinary metabolite, and a geroprotector.

Astragalin is a multifaceted phytochemical with broad and diversified pharmacological applications such as anticancer, anti-inflammatory, antioxidant, neuroprotective, antidiabetic, cardioprotective, antiulcer, antifibrotic, and antiosteoporotic properties [20]. Astragalin exhibits high antioxidant activity by scavenging radicals as well as inhibiting pro-oxidant enzymes and activating antioxidant enzymes. It was demonstrated that kaempherol-3-O-β-d-glucoside has a very good curative effect on cancer, and shows a superior pharmacological effect compared with quercetin.

In the rest of the flavonoid subgroups, the quantities of the metabolites detected are not high, with the only exception being flavanone naringenin recovered by scCO_2_ + ethanol in *G. viscosum* flowers. Its amount is the second among all flavonoids recuperated by that technique, being lower only than kaempferol, and slightly higher but commensurable with quercetin. On the other hand, its amount is over 172-fold higher than the quantity registered in the flower extract of Soxhlet ethanol, and the two-phase solvent. The other compound present in relatively high amount is luteolin, its quantities in the flowers being much higher than those of the two conventional techniques, following the pattern of naringenin.

Myricitrin, naringenin and luteolin are also powerful antioxidants. Myricitrin exhibits antitumor and hepatoprotective properties, while shows strong anti-inflammatory and antioxidant activities, and is beneficial for the treatment of obesity, diabetes, hypertension, and metabolic syndrome. Luteolin possesses antioxidative, antitumor, and anti-inflammatory properties, and exhibits cardiac protective effects.

Finally, the occurrence of two unique caffeoyl-D-glucaric acid derivatives, namely leontopodic acids A and B, is yet another very important characteristic of *G. viscosum*. Though the presence of leontopodic acids A and B in some *Gnaphalium* species, and in particular in five European members of the genus habitual to the Alps, was earlier registered [17], still, as far as we are aware, this is the first work which reports the presence of the two acids in *G. viscosum*. That is a very important finding as, until recently, those acids were predominantly associated only with the protected and difficult to cultivate alpine species *Leontopodium alpinum* (edelweiss) [21,22]. Discovering new sources for those highly potent antioxidants is of considerable importance; moreover, when those resources, such as *G. viscosum*, are abundant and in practice not limited by specific cultivation requirements, or inclusion in protected species lists, etc.

The largest amount of leontopodic acid B was detected in the stem extracts recovered by the two-phase solvent extraction—the quantity being approximately 8- to 10-fold higher than that registered in the flowers and leaves, respectively. The amounts of acid B recovered by Soxhlet ethanol and scCO_2_ + ethanol are orders of magnitude lower than those recuperated in all three extracts of the two-phase solvent. Additionally, the highest amounts of the acid are detected not in the stems but in the leaves for both techniques. With regard to leontopodic acid A, again, the two-phase solvent is the best performer, with the amount in the leaves being the highest, a tendency not observed for Soxhlet ethanol, for which the largest quantity is found in the stem extracts. The acid is not detected in any of the three *G. viscosum* extracts recovered by scCO_2_ + ethanol. Obviously, water/methanol and methyl tert-butyl ether/methanol, which are applied as solvents in the two-phase extraction, exhibit much higher selectivity to leontopodic acids A and B. Those findings are corroborated by the LC–HRAM data, which confirmed that *G. viscosum* stems are a rich source of leontopodic acid B when compared to the flowers and leaves. Soxhlet ethanol does not exhibit good selectivity towards glucaric acid derivatives, which applies even more strongly to scCO_2_ + ethanol extraction.

Leontopodic acids A and B are known for their strong antioxidant potential, anti-inflammatory, antiaging, memory improving, etc., effects. They also possess DNA-protecting properties, help resisting hepatitis virus, protect the liver, and have greater antioxidant potential than alpha-tocopherol (a form of vitamin E). Moreover, because of their beneficial effects on stimulating several key genes and proteins responsible for epidermal protection, the acids are essential in cosmetic formulations.

When analyzing the results obtained, some general observations can be made: it is the usual assumption that because of the presence of unsaturated bonds, polyphenols are heat sensitive and can be oxidized at higher temperatures, particularly when the recovery process is over extended period. In view of this, it would have been expected that scCO_2_ + ethanol would perform better when compared for example to Soxhlet ethanol, taking into consideration the lower temperatures and much shorter extraction times applied. While that might be true for some of the compounds (kaempferol being one such example), for others it is far from so—see chlorogenic acid, and of course astragalin. A possible explanation might be that for certain secondary metabolites, the combined effect of lower temperature, shorter time and a low amount of co-solvent used is negative. As mentioned briefly previously, that assumption is corroborated when the respective quantities in the leaves recovered with either scCO_2_ + 10 or 5% ethanol are compared.

Consequently, it is only fair to conclude that temperature influences a phenolic compound sensitivity in a complex and not straightforward way, and depends on numerous factors, among those physicochemical and biochemical characteristics and type of the particular metabolite, and lastly, but very importantly, on the nature of the plant matrix.

In summary, it should be underlined that the analyses performed have brought to light *G. viscosum* capabilities as a master chemist able to synthesize and store important and highly effective secondary phytochemicals, some of which are unique, with considerable potential for broad and diversified applications in the manufacturing of pharmaceuticals, food additives, cosmetics formulations, etc., targeted at human benefit.

#### 3.2.2. Total Phenolic Content (TPC) and Antioxidant Activity (AA)

Table 5 shows that the flower scCO_2_ + ethanol extract exhibits the highest phenolic content, followed by those of the two-phase solvent and Soxhlet ethanol. Flower extracts, are, however, an exception, since the TPC of the leaves and stems of the two-phase solvent extracts are higher than both Soxhlet ethanol and scCO_2_ + ethanol, the latter being the lowest of the three.

As known, TPC only measures total phenols in the extracts without any identification of the compounds. Additionally, since phenolics show higher affinity towards polar solvents, obviously, the viable explanation of the TPC trends observed is that the solvents applied by the two-phase solvent (water/methanol, and methyl tert-butyl ether/methanol) are more powerful and perform better than ethanol. The latter holds particularly for the scCO_2_+ ethanol extraction, for which the low amount of ethanol used when compared to Soxhlet was not enough to recuperate all phenolic compounds available. Hence, the TPC values calculated for the scCO_2_ + ethanol leaf and stem extracts are 3–9-fold lower than those for the two-phase solvent and Soxhlet ethanol.

The fact that the TPC calculated for the flower extracts is the highest for the three techniques is expected, as the flowers are richer in certain polyphenolic acids (e.g., chlorogenic and gentisic acids mentioned above), as well as in some prominent members of the flavonoids (astragalin, etc.), when compared to the leaves and stems of *G. viscosum*.

With regard to the DPPH free radical scavenging activity and ABTS radical scavenging assay, the same trend as for the TPC is observed; however, this time, there are no exceptions, and the pattern is very clear—the two-phase solvent extraction performs the best, followed by Soxhlet ethanol and scCO_2_ + ethanol. Thus, the activity towards the DPPH radical decreased in the order flowers > leaves > stems for the two-phase solvent extraction and scCO_2_ + ethanol, and flowers > stems > leaves for Soxhlet ethanol (the activity of leaf and stem extracts; however, this is almost commensurable), while for the AA by ATBS, the first order is confirmed with no exceptions.

It can be speculated that certain components extracted were stronger radical scavengers than the rest. Consequently, though they in general present in lower amounts in the extracts of the two-phase solvent when compared to those of Soxhlet ethanol (leontopodic acid B being a remarkable exception), such components can influence and exert a positive influence on the AA.

Moreover, the extracts examined were more effective in DPPH than ABTS radical scavenging (as clearly demonstrated by the IC50 values calculated). That can be a result of the complexity, polarity and chemical properties which could lead to diverging bioactivity. Consequently, the observation reported by some authors that certain compounds might exhibit high scavenging activity in one assay while concurrently lower activity in the other assay [23,24] is supported by our results.

#### 3.2.3. GC–FID

GC–FID analyses of *G. viscosum* leaves and flower extracts obtained by Soxhlet *n*-hexane and neat scCO_2_ were also performed. In what follows, the results for selected leaf extracts are presented in Table 6. The fatty acid profiles determined for the flower extracts are similar, with some not very substantial deviations.

The FAME analysis results for the Soxhlet *n*-hexane and neat scCO_2_ exhibit qualitatively similar fatty acid profiles with the predominant presence of saturated fatty acids (SFAs), with chain lengths of 12–24 carbon atoms (Table 6). Furthermore, a limited number of certain mono-, di- and polyunsaturated fatty acids (MUFAs, DUFAs, and PUFAs, respectively) were also detected, namely palmitoleic, linoleic and γ linolenic acids. Still, it should be noted that neither of the extracts examined contained the three acids—for example, only one of them contained palmitoleic acid in a relatively small amount, linoleic acid was found in all four extracts, while PUFA γ linoleic acid was found in only two of them.

The results also demonstrate the influence of the two techniques and, in further detail, reveal the effect of scCO_2_ extraction process operating conditions on the FAME composition. Thus, as mentioned above, SFAs are the dominant ones, with the lowest percentage being registered in the extract recovered by Soxhlet *n*-hexane, while the highest—is in the extract of scCO_2_ at *T* = 40 °C, *p* = 40 MPa. The latter is practically commensurable with that of the extract recuperated at the highest temperature and pressure applied, in which the over 2.5-fold lower percentage of palmitic acid is compensated by the relatively high percentage of arachidic acid, which is not detected in the extract recovered at the lower temperature and pressure.

With regard to unsaturated fatty acids, the best performer is Soxhlet + *n*-hexane, the second best is scCO_2_ at *T* = 60 °C and *p* = 40 MP. In both extracts, linoleic and γ linolenic acids are detected, with the respective percentages in the two extracts deviating by not very large margins. Hence, a viable assumption can be that in the case of scCO_2_ extraction higher temperature and lower pressure promote the recovery of unsaturated fatty acids.

Additional useful information can be obtained if the ratio (PUFA:SFA) is calculated for each extract presented in Table 6.

On the one hand, for a plant extract to be considered as a suitable source for biofuels production, in addition to other important requirements, its (PUFA:SFA) should be low. On the other hand, however, the PUFA:SFA ratio is among the important parameters currently used to assess nutritional quality of foods. According to the World Health Organization (WHO), the PUFA:SFA ratio should be above 0.4 in the human diet in order to reduce the risk of developing cardiovascular and other chronic diseases [25]. The PUFA:SFA ratios, which were calculated only for the two *G. viscosum* leaf extracts containing γ linolenic acids, show that the Soxhlet *n*-hexane one is the higher of the two but not by an order of magnitude, and complies with the WHO requirements.

A different FAME profile was exhibited by the flower extracts at the lowest pressure applied in the scCO_2_ extraction (*p* = 30 bar) and *T* = 40 °C. SFAs are the dominant ones, with the palmitic acid relative presence being the highest (41. 6%). Still, it is lower than the relative presence of the only unsaturated fatty acid detected—the DUFA linoleic acid with 43.99%, respectively.

If the FAME profile of the flower extract is compared with that of the leaves at the same operating conditions (*T* = 60 °C, *p* = 50 MPa), again the SFAs are the dominant ones, with capric and lauric acids also present, though with low relative percentages. Two unsaturated fatty acids are detected, palmitoleic and linoleic acid. The latter is the most abundant, with a percentage higher than that registered in the leaves (34.42%). In complete analogy with the leaves, the PUFA γ linolenic acid is not found.

## 4. Conclusions

*G. viscosum* is a widely distributed plant with enormous potential. In this study, three different extraction techniques were applied to recover highly potent metabolites from the plant’s flowers, leaves, and stems. Advanced analysis techniques such as LC–MS/MS, and LC–HRAM were used to characterize the extracts and important representatives of phenolic acids (hydroxycinnamic and caffeoylquinic acid derivatives), hydroxybenzoic acid derivatives, and flavonoids, flavones, flavan-3-ols, flavanones and proanthocyanidins were determined. For the first time, in the present study, the powerful antioxidants leontopodic acids A and B were identified and quantified in the species. Additionally, the fatty acid profile of the extracts obtained was determined by applying GC–FID. This extensive experimental work for extraction, characterization, and evaluation of the antioxidant capacity of *G. viscosum* extracts represents a significant and important contribution in pushing forward the knowledge boundaries about the potential of this biomass and the prospects for its efficient valorization.

## Figures and Tables

**Table 1:** Soxhlet extraction yields of *G. viscosum* leaves, flowers and stems using different solvents.

Solvent	Extraction Yield (wt%)
**Leaves**	
Ethanol	17.18 ± 0.84
Ethyl Acetate	7.27 ± 0.36
*n*-Hexane	3.99 ± 0.21
**Flowers**	
Ethanol	12.23 ± 0.56
Ethyl Acetate	3.96 ± 0.19
*n*-Hexane	3.48 ± 0.17
**Stems**	
Ethanol	8.20 ± 0.38
Ethyl Acetate	2.88 ± 0.14
*n*-Hexane	1.81 ± 0.09

Extraction yield expressed in wt% (mean ± standard deviation).

**Table 2:** LC–HRAM analyses of *G. viscosum* flowers, leaves and stems extracts obtained by the two-phase solvent extraction.

	3-O-Caffeoylquinic (Chlorogenic) Acid	5-O-Caffeoylquinic (Neo-Chlorogenic) Acid	Glucaric Acid Derivatives	
			Leontopodic Acid A	Leontopodic Acid B
	ng/mg			
Flowers	4710.0	286.3	12.9	167.2
Leaves	3366.9	198.8	8.1	141.1
Stems	2752.8	98.7	14.1	1132.7

Relative standard deviation (RSD) = ±3.4%.

**Table 3:** LC–MS/MS analysis of phenolic compounds in selected *G. viscosum* flowers, leaves and stems extracts obtained by the two-phase solvent and Soxhlet ethanol extractions.

Compound	Two-Phase Solvent Technique			Soxhlet EtOH		
	Flowers	Leaves	Stems	Flowers	Leaves	Stems
ng/mg						
Phenolic Acids						
Hydroxycinnamic and caffeoylquinic acid derivatives						
caffeic acid	3.15	4.28	1.95	5.99	17.21	9.24
*o*-coumaric acid	2.21	5.03	4.35	10.61	16.09	4.65
*p*-coumaric acid	0.14	0.16	0.19	0.43	0.31	0.25
*m*-coumaric acid	0.05	0.81	0.18	0.34	0.39	0.89
ferulic acid	5.04	4.77	4.62	14.01	19.23	6.11
cinnamic acid	2.63	11.14	3.48	3.99	12.53	4.56
3-O-caffeoylquinic (chlorogenic) acid	3618.95	2116.35	1925.44	5668.11	2421.34	2041.15
Hydroxybenzoic acid derivatives						
gallic acid	6.24	1.71	5.94	5.94	1.48	0.57
vanillic acid	69.66	64.21	46.35	10.23	21.97	1.89
ellagic acid	2.79	11.59	1.75	9.78	2.98	7.96
gentisic acid	351.82	181.58	52.26	866.72	465.26	284.73
protocatechinic acid	55.46	53.48	14.09	35.58	33.44	0.23
*o*-hydroxybenzoic acid	22.95	19.38	7.22	0.33	2.35	0.93
*m*-hydroxybenzoic acid	4.03	4.93	0.96	7.25	6.73	4.26
syringic acid	5.46	9.11	6.73	12.92	16.12	3.02
3-OH-4-methoxybenzoic acid	46.41	52.70	263.47	8.58	46.51	24.04
Flavonoids						
Flavonols						
quercetin	331.41	39.93	17.41	450.27	92.48	30,96
myrecitrin	790.80	200.78	56.72	1288.26	398.57	439.65
myrecitin	n.d.	n.d.	n.d.	n.d.	n.d.	n.d.
rutin	4.80	1.46	0.98	5.91	38.25	90.06
resveratrol	n.d.	n.d.	n.d.	n.d.	n.d.	n.d.
kaempferol	798.86	82.59	13.79	1106.91	72.32	39.68
kaempferol-3-O-glycoside	45999.92	6589.52	1207.40	76129.67	7993.67	9827.97
kaempferitin	n.d.	n.d.	n.d.	n.d.	n.d.	n.d.
fisetin	7.93	2.17	1.01	21.12	2.37	2.32
Flavones						
luteolin	6.24	1.71	0.44	5.94	1.48	0.57
apigenin	77.97	7.22	1.02	81.78	4.47	2.39
Flavan-3-ols						
catechin	n.d.	n.d.	n.d.	n.d.	n.d.	n.d.
epicatechin	0.21	0.30	0.06	1.31	0.29	0.13
epigallocatechin	3.74	n.d.	n.d.	n.d.	n.d.	n.d.
epigallocatechin gallate	0.00	n.d.	0.01	0.01	0.02	0.02
epicatechin gallate	0.19	0.22	1.91	0.22	0.12	0.16
Flavanones						
hisperidin	2.43	0.028	0.03	0.71	0.29	0.42
naringenin	11.02	2.08	0.58	19.91	2.52	1.95
Proanthocyanidins						
procyanidin B1	7.14	7.15	7.14	7.17	7.13	7.14
procyanidin B3	n.d.	n.d.	3.19	n.d.	4.86	n.d.
Caffeoyl-D-glucaric acid derivatives						
Leontopodic acid A	29.55	211.28	119.13	6.62	16.80	21.93
Leontopodic acid B	134.94	95.02	1011.05	2.23	71.36	4.37

Relative standard deviation (RSD) = ±2.3%. n.d—Not detected.

**Table 4:** LC–MS/MS analysis of phenolic compounds in selected *G. viscosum* flowers, leaves and stems extracts obtained by scCO_2_ + 10% ethanol for the flowers, leaves and stems, and 5% ethanol for the leaves.

Compound	Flowers	Leaves	Stems	Leaves (5% EtOH)
[ng/mg]				
Phenolic Acids				
Hydroxycinnamic and caffeoylquinic acid derivatives				
caffeic acid	1999.37	604.53	113.46	39.30
*o*-coumaric acid	173.29	5.59	36.91	4.95
*p*-coumaric acid	1.38	0.38	0.36	0.42
*m*-coumaric acid	10.32	2.30	1.74	1.04
ferulic acid	206.24	39.26	22.39	12.88
cinnamic acid	9.94	21.07	4.85	15.31
3-O-caffeoylquinic (chlorogenic) acid	62.49	60.89	16.64	2.88
Hydroxybenzoic acid derivatives				
gallic acid	6.73	4.07	10.46	0.21
vanillic acid	772.21	162.03	51.54	46.25
ellagic acid	24.70	3.02	1.69	1.19
gentisic acid	1091.63	184.44	49.51	14.93
protocatechinic acid	32.55	24.56	1.21	1.33
*o*-hydroxybenzoic acid	174.88	41.38	17.79	8.04
*m*-hydroxybenzoic acid	47.90	19.33	5.39	3.99
syringic acid	160.85	50.37	33.33	17.08
3-OH-4-methoxybenzoic acid	726.99	138.40	48.99	45.90
Flavonoids				
Flavonols				
quercetin	3215.37	206.14	123.49	21.76
myrecitrin	307.14	32.38	5.62	0.95
myrecitin	n.d	n.d	n.d	n.d
rutin	17.33	11.49	5.13	1.98
resveratrol	n.d.	n.d.	n.d.	n.d.
kaempferol	10731.82	383.51	99.97	33.20
kaempferol-3-O-glycoside	2574.92	221.52	45.81	8.49
kaempferitin	n.d	n.d	0.01	n.d
fisetin	30.46	3.55	0.36	0.14
Flavones				
luteolin	434.97	14.99	4.89	1.54
apigenin	15.01	0.19	0.19	0.17
Flavan-3-ols				
catechin	n.d.	n.d.	n.d.	n.d.
epicatechin	1.70	0.01	n.d.	0.00
epigallocatechin	1.35	0.11	0.01	0.02
epigallocatechin gallate	0.10	n.d.	n.d.	n.d.
epicatechin gallate	0.12	0.02	0.01	0.01
Flavanones				
hisperidin	1.19	0.71	0.89	0.76
naringenin	3294.13	83.91	13.42	23.06
Proanthocyanidins				
procyanidin B1	n.d.	n.d.	n.d.	n.d.
procyanidin B3	n.d.	n.d.	n.d.	n.d.
Glucaric acid derivatives				
Leontopodic acid A	n.d.	n.d.	n.d.	n.d.
Leontopodic acid B	n.d.	34.93	0.39	n.d.

Relative standard deviation (RSD) = ±2.3%. n.d—Not detected.

**Table 5:** Total phenolic content, antioxidant activity and IC_50_ by DPPH and ABTS of *G. viscosum* flowers, leaves and stems extracts.

Extraction Method	TPC	DPPH		ABTS	
Two-Phase Solvent	Quercetin eq. [µg/mg]	Trolox eq. [mM]	IC_50_ mg Extract	Trolox eq. [mM]	IC_50_ mg Extract
flowers	130.87	12.72	1.34	9.48	2.27
leaves	95.70	9.25	2.72	6.07	4.11
stems	76.83	4.93	2.46	4.01	7.01
**Soxhlet ethanol**					
flowers	113.28	6.85	0.68	5.44	1.24
leaves	69.54	3.76	0.96	3.27	2.02
stems	58.81	4.08	1.96	2.15	3.22
**scCO_2_ + 10% ethanol**					
flowers	162.11	1.64	3.15	2.71	36.08
leaves	36.96	0.65	18.45	1.14	10.98
stems	6.39	0.25	90.10	0.35	48.20
Leaves 5% EtOH	8.05	0.22	126.42	0.55	25.93

Relative standard deviation (RSD): RSD_DPPH_ = ±3.01%; RSD_ATBS_ = ±3.95%; RSD_IC50_ = ±1.6%.

**Table 6:** Fatty acid composition from FAME GC–FID analysis of selected *G. viscosum* leaves extracts recovered by different extraction methods, expressed as the relative percentage of total fatty acids identified.

Fatty Acid	Soxhlet *n*-Hexane		scCO_2_, *T* = 40 °C, *p* = 40 MPa		scCO_2_, *T* = 60 °C, *p* = 40 MPa		scCO_2_, *T* = 60 °C, *p* = 50 MPa	
	%	Str. Dev.	%	Str. Dev.	%	Str. Dev.	%	Str. Dev.
Lauric acid, 12:0	n.d.		n.d.		0.40	0.05	n.d	
Myristic acid, 14:0	4.68	0.48	5.67	0.97	3.04	0.40	3.19	1.2
Palmitic acid, 16:0	22.77	1.13	49.42	9.59	31.57	5.53	19.36	6.35
Palmitoleic acid, 16:1	n.d.		n.d.	-	n.d.		4.46	2.62
Stearic acid, 18:0	13.67	1.35	n.d.		n.d		n.d.	
Linoleic acid, 18:2	20.42	3.44	31.34	7.44	22.25	11.57	27.35	4.83
Arachidic acid, 20:0	n.d		n.d.	-	11.12	0.81	33.32	15.8
γ-Linolenic acid, 18:3	25.48	8.38	n.d.		22.05	6.17	n.d.	
Behenic acid, 22:0	5.42	2.20	7.15	1.79	4.83	0.94	6.52	0.7
Lignoceric acid, 24:0	7.55	3.60	6.41	1.56	4.74	1.59	5.81	1.28
SFA	54.1		68.66		55.7		68.18	
MUFA	n.d.		n.d.		n.d.		4.46	
DUFA	20.42		31.34		22.25		27.35	
PUFA	25.48		n.d.		22.05		n.d.	
PUFA:SFA	0.471		0.0		0.386		0.0	

n.d—Not detected.

## Data Availability

Not applicable.
